# Is performance in goal oriented head movements altered in patients with tension type headache?

**DOI:** 10.1186/1471-2474-15-179

**Published:** 2014-05-26

**Authors:** Andrée-Anne Marchand, Vincent Cantin, Bernadette Murphy, Paula Stern, Martin Descarreaux

**Affiliations:** 1 Université du Québec à Trois-Rivières, 3351 boul. des Forges, C.P. 500 Trois-Rivières, Québec G9A 5H7, Canada; 2 University of Ontario Institute of Technology, 2000 Simcoe St North, Oshawa, Ontario L1H 7 K4, Canada; 3 Canadian Memorial Chiropractic College, 6100 Leslie Street, Toronto, Ontario M2H 3 J1, Canada

**Keywords:** Tension type headaches, Motor control, Cervical spine, Kinesthetic sense

## Abstract

**Background:**

Head repositioning tasks have been used in different experimental and clinical contexts to quantitatively measure motor control performance. Effects of pain on sensorimotor control have often been described in various musculoskeletal conditions and may provide relevant information with regard to potential mechanisms underlying tension-type headaches. The purpose of the current study was to compare the performance of patients with tension-type headache and healthy participants in a cervical aiming task using the Fitts’ task paradigm.

**Methods:**

Patients with tension-type headache and healthy controls were compared in a cervical aiming task. Participants were asked to move their head as quickly, and precisely as possible to a target under various experimental conditions. Dependent variables included movement time, variable error, constant error and absolute error.

**Results:**

As predicted by Fitts’ law, decreasing target size and increasing head rotation amplitudes yielded longer movement times in both groups. Participants with tension-type headache, when compared to healthy participants showed a significant increase in both constant and absolute errors for each of the four conditions.

**Conclusion:**

Decreased motor performance was observed in participants with tension-type headache, likely due to altered motor control of the neck musculature. Future research is warranted to investigate the clinical aspect related to decrease in motor performance.

## Background

Tension-type headache (TTH), is defined as episodes of bilateral cephalic pain, characterized as “band-like” pressing or tightening sensation of mild to moderate intensity which may or may not be associated with pericranial tenderness on manual palpation [1]. According to a review of population based studies, the worldwide point prevalence of headache can be estimated at 47%, of which 38% is attributed to TTH [2]. This is the most common type of primary headache with lifetime prevalence in the general population ranging from 30 to 86% [1,3]. Furthermore, frequent episodic and chronic TTH are reported significantly more frequently in women than men [4], causing greater disability and leading patients to seek care [5].

Despite advances in the understanding of TTH, controversy exists in the literature regarding its aetiology and best management strategies [6,7]. The diagnosis of TTH is primarily based on clinical signs and symptoms gathered through history taking. Although pericranial tenderness is considered a facultative criterion to the diagnosis of TTH, there has been no objective physical outcome measure systematically proposed to help clinicians with the diagnosis. Physiological outcome measures are commonly studied in individuals with low back or cervical pain [8,9] and have been used effectively to discriminate healthy controls from symptomatic patients with these conditions [9-11]. Furthermore, a recent scoping review on musculoskeletal physical outcome measures in individuals with TTH has highlighted the scarce use of such data in clinical research [12]. While both myofascial pain detection and tolerance thresholds are consistently decreased in patients with TTH [13] suggestive of central sensitization [14], other commonly used outcomes such as presence of trigger points, muscle cross sectional area, level of muscle activity and cervical range of motion yielded mixed results [12].

Investigations into the effect of musculoskeletal conditions on sensorimotor control suggest that patients experiencing pain develop motor adaptations as a way to alleviate symptoms [15]. It is proposed that if the motor adaptation is excessive or persists past the painful episode, it contributes to the perpetuation or recurrence of pain [15]. Thus, muscular control is at the center of several models that aim to understand motor planning and response in patients experiencing pain [16]. Changes in sensorimotor function have been shown to have profound effects on control of movement in many painful conditions of the musculoskeletal system such as neck pain [17], low back pain [18] and shoulder pain [19]. Alterations in sensorimotor functions include reduced sensory acuity [20], reduced responsiveness to sensory input, reorganisation of the sensory cortex and smudging of motor regions of the brain [21-23], and increased errors in position sense [24]. Such modifications have been observed in the cervical region of patients with whiplash associated disorders and nonspecific subclinical neck pain who displayed decreased kinaesthetic sense when tested in a cervical aiming task [25-27].

Cervical repositioning accuracy tasks measure the ability of the neuromuscular system to actively reposition the head in a given posture after active movements in different planes of movement. It is generally accepted that the receptors primarily responsible for joint position sense are the muscle spindles. Muscle spindles are found in very high density in the deeper cervical muscles of the upper cervical spine such as the sub occipital complex (superior oblique capitis, inferior oblique capitis and rectus capitis posterior major and minor) when compared with lower cervical spine [28,29]. In addition, the relatively low number of mechanoreceptors found in human cervical facet joint capsules suggests a complementary role to that of muscle receptors in the mediation of position sense, particularly at the end of motion ranges [30]. The afferent input derived from muscle, cutaneous and joint receptors along with information from the vestibular and visual systems are all together integrated to build an internal reference frame of the musculoskeletal system and to recalibrate it [30,31]. Consequently, a cervical spine positioning error is considered to mainly reflect disturbed afferent input from the articulations of the neck and muscle receptors [32].

The cervical kinaesthetic task developed by Revel in 1991 and modified by Loudon in 1997 has shown to be a valid and reliable measure of the cervical sensorimotor control [33-35] and has been commonly used in whiplash associated disorders [17,36] and nonspecific neck pain populations [37]. Motor performance of the cervical spine has also been established through the measurement of movement time when engaged in a Fitts' task experiment [37-39]. Fitts' task experiment is derived from the speed accuracy trade-off in target-constrained aiming situations. The Fitts' law states that as target size decreases or as amplitude between targets increases, the movement time will increase linearly [40]. It is considered a reliable descriptor of the information processing demands associated with a variety of aiming movements [41]. Since movement time dictates the type of feedback corrections that are possible and the relative contribution of different movement control mode, increasing the index of difficulty provides information on how sensory information (target size and distance) is used to generate a motor response. The Fitts’ task allows the assessment of motor skill performance and is particularly well suited for clinical studies, as performance during the task is resistant to learning effects [41].

A recent study has highlighted that chronic neck patients increase their movement time and use alternative control strategies in order to reduce response variability and be as accurate and consistent as possible in their performance [37].

Therefore, the purpose of the current study was to compare the performance of patients with TTH and healthy participants in a cervical aiming task using the Fitts’ task paradigm.

Given the sensorimotor changes observed in neck pain populations under varying experimental models and the proposed implications of the cervical region to explain the pathophysiology of TTH, it is hypothesized that patients with TTH have similar sensorimotor deficits and that theses will be positively correlated to clinical parameters.

## Methods

### Participants

All participants (N = 33; female = 85%) were recruited by convenience from the university community and employees. Given that there was no data available to conduct a standard sample size calculation, the sample size estimate was based on a similar study looking at kinematics in a head reorientation task using the Fitts' paradigm[37]. Inclusion criteria for TTH patients consisted of fulfilling the second version of the International Headache Society (IHS) classification criteria for primary tension type headache and to experience headache at least once per month. Participants of the control group were included on the basis that they were healthy and had no previous history of headaches. Inclusion and exclusion criteria are listed in Table 1. In order to take part to the experimental session the participants were screened for the presence of potential confounders such as an ongoing episode of headache, having received treatment in the past 72 hours, and wearing clothes that cover the neck. After this initial screening, participants provided written informed consent prior to demographic data collection and laboratory testing as approved by the Université du Québec à Trois-Rivières human ethics committee (certificate # CER-12-181-06.23).

**Table 1:** Inclusion and exclusion criteria

**Inclusion criteria**	**Exclusion criteria**
**Tension-type headache subjects**	Rheumatoid arthritis
Fulfilling criteria of the International Headache Society (IHS) for TTH:	Impairment of vestibular system
	Visual impairment
Headache lasting from 30 minutes to 7 days	Hearing impairment
Headache has at least two of the following characteristics:	Other types of headache
1. bilateral location	Present episode of headache
2. pressing/tightening (non-pulsating) quality	Neck pain as main complaint
3. mild or moderate intensity	Neck trauma
4. not aggravated by routine physical activity such as walking or climbing stairs	Active site of pain
Both of the following:	Chronic pain syndrome
1. no nausea or vomiting (anorexia may occur)	Whiplash associated disorders
2. no more than one of photophobia or phonophobia	
Not attributable to another disorder	
Experiencing at least 1 episode/month	
**Control subjects**	
No headache	

### Outcome measures

Participants in the experimental group were questioned about the frequency of headache episodes and were asked to score the intensity of their typical headache prior to the experimental session using a 100 mm visual analog scale (VAS). A VAS score was also obtained from all participants after the experimental task to evaluate if any pain developed during the experimental session. The Neck disability index (NDI), the State-Trait Anxiety Inventory (STAI) and the Headache Impact Test (HIT-6) were used to respectively quantify neck disability, the strength of an individual’s feelings of state and trait anxiety across typical situations, and the effect that headaches have on daily ability function. The French versions of the questionnaires which were used have all been validated [42-44]. Cervical ranges of motion were also quantified using a cervical range of motion (CROM) goniometer to ensure participants’ ability to complete the experimental task [45]. Ranges of motion in all planes were measured twice and the mean value was recorded. The range of motion assessment was performed to ensure that all participants could reach 50° of rotation and to identify potential differences between the two groups.

### Aiming task

For this study, the Fitts’ task paradigm was chosen to ensure that an optimal challenge point would be met in at least one of the experimental conditions. Index of difficulty variation [40] is often used to alter the functional level of a task to enhance differences between groups that may otherwise not be observed [37]. A custom made experimental set-up similar to that used in previous studies was developed to assess cervical kinesthetic performance [37]. A laser pointer mounted on a helmet was secured to the head and tightened enough to avoid extra movement yet still be comfortable for the participants. In a quiet and dimly lit room, participants were seated on a chair with back rest facing a black semi-circular board at a radial distance of 1.75 m. The seat height was adjusted so that the participants had their eyes level with the targets. Participants were instructed first to close their eyes and to produce a few non-maximal flexion, extension and rotation head movements and then assume a neutral final position. This position was used as the starting point reference (0°) for every subsequent trial with the laser pointer oriented in the center of the board. Reference positions (25° and 40°) were also recorded for each of the conditions in order to determine movement time, directional error, absolute error and variable error. The instructions provided at this point were standardized and always provided similarly for all participants. Participants were told to rotate their head as quickly and precisely as possible to the target, and then back to the neutral head position. Participants were asked not to attempt to correct the movement once it was initiated (this was to ensure that the movement was made without any correction based on sensory feedback during the pointing task). Four different conditions were represented by two target sizes (8 cm and 12 cm diameter), and two rotation amplitudes (25° and 40° from the neutral head position). The index of difficulty were calculated using the Fitts’ law formula: [log_2_(2A/W)]; where A represents movement amplitude, and W represents target width. The arc length corresponding to the axial rotation motion was calculated to obtain each index of difficulty. Index of difficulty of each of the four conditions was: condition 1 (3.67); condition 2 (4.25); condition 3 (4.35); condition 4 (4.93). Due to the repetitive nature of the experimental task and high concentration demand, arrangements had to be made to avoid any upsurge of headache. Therefore, participants were randomly asked to perform the four conditions on either right or left side of the board only. All conditions were conducted in a random order to control for potential sequence order effects. Each participant was allowed 15 trials with eyes opened to familiarize with a given condition before the recording period consisting of 10 trials with eyes closed. Each trial was performed successively with approximately 5 seconds between each repetition. This process was repeated for each of the four conditions. Kinematic data were collected using an active marker motion analysis system (Optotrak Certus, Northern Digital, Waterloo, ON, Canada). Three light-emitting diodes positioned on the helmet were used to create a vector. Angular displacement of the head in the transverse plane was calculated by subtracting the initial angular position from the final angular position of the vector. Kinematic data was collected at 100 Hz, and was low-pass filtered using a dual-pass, fourth-order Butterworth filter with a cut-off frequency set at 5 Hz.

### Data analysis

Dependent variables included movement time (MT), variable error (VE), constant error (CE) and absolute error (AE). Movement time is represented by the time required to move from resting position to the target. VE measures the inconsistency in movement outcome. It represents the difference between the participant’s movement amplitude score on each trial and his or her own average score. CE represents the positive or negative difference between the amplitude reached and the target. A positive CE corresponds to overshooting the target while a negative CE corresponds to undershooting the target. AE represents the average absolute deviation (without regard to direction) between the participant’s responses and the target. These four variables are commonly used as markers of motor performance during aiming task [46].

### Statistical analysis

The statistical analysis was performed with Statistica data analysis software system, version 10 (StatSoft, Tulsa, OK, USA). Normality of distribution for every dependent variable was assessed with the Kolmogorov-Smirnov test and through visual inspection of data. T-tests for independent samples were conducted for baseline values of continuous variables. All dependent variables (MT, CE, VE and AE) were found to be normally distributed and were therefore submitted to a mixed model ANOVA, with Movement Amplitude (25° and 40°) and Target Size (8 cm and 12 cm) as within-subject factors and Group as between-subject. Whenever a main or interaction effect was observed, post hoc comparisons were made using Tukey’s test. Polynomial contrasts were also conducted to test for the linear trend in movement time (linear relationship between movement time and target index of difficulty). Effect size estimates were calculated by partial eta-squared (ηp2; 0.01 = small effect; 0.06 = medium effect; 0.14 = large effect). In order to assess the association between clinical status and motor performance, simple correlation tests were performed separately for each clinical outcome (Intensity and Frequency of pain, HIT-6, NDI) and dependent variable (MT, CE, AE, VE). Statistical significance was set, for all analyses, at p < 0.05.

## Results

A total of 33 participants were recruited for this study. The tension type headache group (n = 16) included individuals with either episodic or chronic tension type headache. Seventeen healthy participants without any prior history of headache or with less than two non disabling headache episodes per month and which had never been given a medical diagnosis were included in the study to form the control group. T-tests for independent samples revealed that both groups were comparable for age, weight, height and both STAI scores (all p > 0.05). A significant difference in baseline pain scores (p = 0.01), frequency of episodes (p > 0.0001), neck disability scores (p = 0.0003) and headache impact scores (60.0 ± 9.2 for the TTH group, N/A for control) was observed between the two groups. The total cervical range of motion for rotation and lateral flexion was similar in both groups (p > 0.05) whereas total range of motion for cervical flexion-extension (125,7° ± 16,1°) was significantly decreased in the TTH group (p = 0.02). Participants’ baseline characteristics and cervical range of motion are presented in Table 2.

**Table 2:** Participants’ baseline characteristics and cervical range of motion

	**Group 1 (healthy controls)**		**Group 2 (patients with TTH)**		
	**Mean**	**SD**	**Mean**	**SD**	**p**
Age (years)	30.4	7.9	28.1	8.1	0.40
Frequency/month	0.4	0.4	7.5	4.8	<0.001
Pain intensity (cm)	3.6	2.2	5.4	1.3	0,01
Weight (kg)	65.1	12.2	67.2	11.5	0.60
Height (cm)	169.1	8.4	166.3	7.6	0.32
NDI (/50)	1.9	4.1	11.6	8.9	<0.001
HIT-6 (36–78)	0	-	60.0	9.1	0
STAI – State (20–80)	49.2	2.8	48.1	4.1	0.38
STAI – Trait (20–80)	47.9	4.2	47.1	4.2	0.56
ROM flex-ext (deg)	140.8	20.2	125.7	16.1	0.02
ROM rotation (deg)	146.8	18.4	138.3	16.1	0.16
ROM lat. flexion (deg)	91.8	17.2	90.1	13.9	0.75

**Figure 1:**
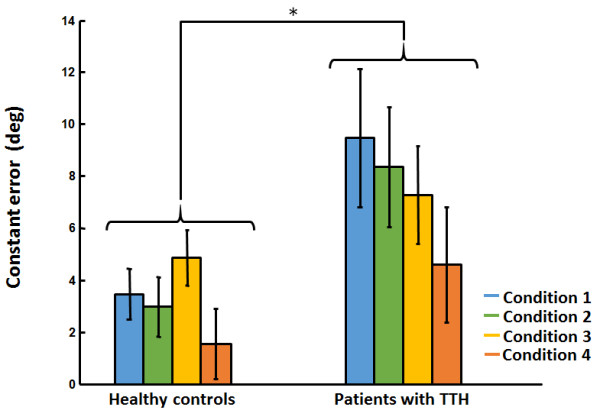
Mean (SD) constant errors for each group throughout all conditions.

**Figure 2:**
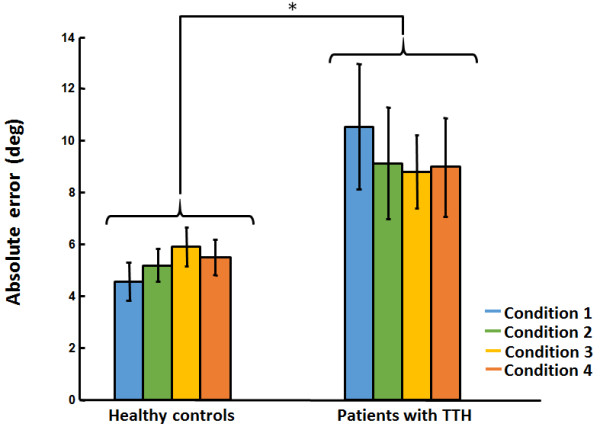
Mean (SD) absolute errors for each group throughout all conditions.

The ANOVA revealed, as predicted by Fitts’ law speed-accuracy trade-off principle, that decreasing target size and increasing head rotation amplitudes (increasing index of difficulty) yielded longer MT in both groups (see Table 3). However, no group difference could be identified (p > 0.05). Polynomial contrasts confirmed the significant linear trend (p < 0.0001).The ANOVA also revealed a significant group effect for both constant and absolute errors whereas no Group differences could be identified in movement time and variable error (p > 0.05). Patients with TTH showed significantly increased CE (F(1,31) = 4.22, p = 0.048, ηp2 = 0.12) and AE (F(1,31) = 5.18, p = 0.03, ηp2 = 0.14). Mean (SD) CE and AE for each group throughout all conditions are presented in Figures 1 and 2.

**Table 3:** Mean (SD) values for all dependant variables

		**Group 1 (healthy controls)**		**Group 2 (patients with TTH)**	
		**Mean**	**SD**	**Mean**	**SD**
Movement time (ms)	Condition 1	551	45	567	37
	Condition 2	518	37	562	33
	Condition 3	592	42	594	39
	Condition 4	615	41	646	49
Constant error (deg)	Condition 1	3.4	0.9	9.4	2.6
	Condition 2	2.9	1.1	8.3	2.3
	Condition 3	4.8	1.0	7.2	1.8
	Condition 4	1.5	1.3	4.5	2.2
Absolute error (deg)	Condition 1	4.5	0.7	10.5	2.4
	Condition 2	5.1	0.6	9.1	2.1
	Condition 3	5.8	0.7	8.7	1.4
	Condition 4	5.4	0.6	8.9	1.9
Variable error (deg)	Condition 1	2.9	0.2	4.0	0.5
	Condition 2	3.4	0.2	3.4	0.4
	Condition 3	3.1	0.3	3.4	0.3
	Condition 4	3.4	0.2	4.4	1.5

Condition 1: 25°,12 cm; condition 2: 25°, 8 cm; condition 3: 40°,12 cm; condition 4: 40°8 cm

Neither Group x Target size nor Group x Movement amplitude interaction effect were present (p > 0.05) for any of the dependant variables. Mean (SD) values for all dependant variables in patients with TTH and healthy participants are presented in Table 3.

Moderate positive correlations were observed between CE and respectively TTH frequency (r = 0.38, p = 0.03), NDI scores (r = 0.42, p = 0.01) and HIT-6 scores (r = 0.34, p = 0.048). Moderate positive correlations were also identified between AE and TTH frequency (r = 0.40, p = 0.02), NDI scores (r = 0.45, p = 0.009) and HIT-6 scores (r = 0.40, p = 0.048).

## Discussion

The objective of the present study was to compare the motor performance of patients with TTH and healthy participants using a cervical aiming task performed under the Fitts’ law paradigm.

The main findings of this study indicated that patients with TTH show significantly decreased motor performance in an aiming task involving the cervical spine when compared to healthy adult controls. Although participants with TTH were able to reproduce head movements with similar movement time and variability, they were not as accurate as the healthy participants. Interestingly, headache frequency, cervical disability and headache related disability were moderately correlated to both CE and AE indicating that changes in sensorimotor control of the head and neck movement may increase with flare-ups or chronification of TTH. The index of difficulty and movement time relationship observed during the cervical aiming task yielded similar results to those reported in previous Fitt’s task studies involving the cervical spine. Indeed, increasing the difficulty index by modulating target sizes and rotational movement amplitudes yielded increased movement time, consistent with the linear speed-accuracy trade-off principle [37,39,40,47]. In fact, increases in movement time for the most challenging condition yielded a more accurate performance in both groups, although such difference was not statistically significant. Using increasing levels of difficulty forces participants into a compromise where feedback, under difficult circumstances, must be integrated to provide an appropriate response. In the present study, participants performed the task slower in order to meet the precision criteria.

To the best of our knowledge, this study is the first to document cervical sensorimotor performances in individuals with TTH. Studies of head repositioning accuracy conducted in cervicogenic headache and migraine populations have found no difference between symptomatic and control participants [16,48,49]. Distinct pathophysiological features between TTH and other types of headache and the fact that these early studies were not designed to primarily assess motor performance, may in part account for these differences. Although the joint positioning error is commonly tested with the subjects relocating their head to a neutral posture following active cervical movements [17,26] our method appears to be adequate to test sensorimotor dysfunction in patients with cervical musculoskeletal conditions. Modulating the difficulty index of a motor task may prove to be of particular interest to detect subtle deficits in various clinical populations.

The impairment of motor performance in a TTH population, suggests underlying changes in afferent input from the cervical muscles which may only become apparent in conditions where visual input is unavailable or unreliable and vestibular input insufficient to perform the task accurately.

Results of the current study highlight the potential benefit of motor control training rehabilitation protocol in the treatment of patients with TTH such as the one suggested by Van Ettekoven et al. [50]. Given the significant differences observed for all four level of difficulty, a simpler version of the test could be developed to identify and monitor cervical sensorimotor changes in the experimental or clinical setting.

Available ROM may be a limiting mechanical factor influencing motor performance in a cervical aiming task. The values recorded for cervical ROM in the various movement planes were similar to the age-dependent values reported in a healthy population even though the baseline values were significantly different between the TTH group and the healthy participant group for the flexion-extension range [51]. Among the few studies looking at ROM in populations with TTH, decreases in ROM are reported to be variable [12]. As such, previous studies have described active cervical flexion as decreased in patients with TTH and active extension as being similar to healthy controls. Overall, patients with TTH involved in this study presented with significant decreases in both flexion and extension ROM which could not be explained by presence of pain neither by age or gender differences between groups. These differences, however, may have only limited impact during head rotation and cannot explain the decreased motor performance in an aiming task observed in patients with TTH.

It has been suggested that sensorimotor deficits associated with painful conditions could be explained by changes at multiple levels of the motor system leading to altered mechanical behaviour and redistribution of muscles activity with the aim to protect from further pain or injury [15]. Possible central pathophysiological mechanisms of TTH include central sensitization of nociceptive circuits and impaired supraspinal descending inhibitory control of pain [52]. Changes in cervical muscle electromyographic activity have also been described but remain controversial and perhaps a distinctive features of episodic TTH [12]. From an anatomical perspective, preliminary studies have identified fibrous connections travelling through the atlantoaxial interspace [53] which may impact dural tension during movements of the atlanto-occipital and atlanto-axial vertebral joints and result in a variety of clinical manifestations [54]. Whether or not these structures and physiological mechanisms play a significant role in TTH remains to be determined.

### Limitations

In the present study, patients presented with fairly good functional status and a limited number of patients with severe TTH were included (N = 5). Therefore, the participants involved in the present study represent a subgroup of patients with TTH which might explain the lack of clear associations between the clinical parameters and physical outcomes. Higher levels of pain and disability may lead to distinctive features of sensorimotor adaptation which might not have been identified in this study. Furthermore, inclusion of healthy controls with a prior history of headache may tamper the differences identified between the two groups. However, considering that the lifetime prevalence of TTH in the general population can reach up to 86% and is likely to affect most individuals at least once, the participants are estimated to be a fair representation of the general population.

The vestibular system plays an important role in maintaining an accurate representation of self-motion. Indeed, the vestibular system provides a veridical representation of head motion to higher-order centers for the perception of self-motion and spatial memory [55]. Although participants in our study were screened for potential vestibular disorders, we cannot exclude the possibility that the activation of the vestibular system occurred during rapid head movement, therefore contributing to task performance optimization. Future research settings should include perturbation of the vestibular system in order to tease out the contribution of the different sensory inputs.

It is also possible that fatigue or attentional disruption may have played a role in the participants’ performance. However, a recent article by Moore et al. [56] investigating attentional disruption in patients with headache emphasized that headache pain appears to impair general task performance, irrespective of task complexity, rather than specific attentional mechanisms. Thus, in light of these results, we believe that the effect of pain on attention was limited by conducting all experimental sessions when TTH patients were not in an ongoing headache episode.

Due to its nature, a cross-sectional study does not allow for an assessment of the temporality of association. Longitudinal studies will be required to determine which of the TTH symptoms or impaired motor performance came first.

Future research should focus on evaluating the association between different stages of clinical disability and the associated motor adaptations. Identifying clinical and physiological factors involved in the transition from episodic to chronic TTH is a key issue in the development of original conservative approaches. A better understanding of the physiological mechanisms underlying motor adaptations in TTH populations will certainly inform both evaluation and clinical interventions as part of rehabilitation protocols.

## Conclusion

The results from the present study indicate that performance in goal oriented head movement is decreased in individuals with TTH when compared to healthy participants. Peripheral mechanisms described as myofascial nociception and central mechanisms leading to sensitization and inadequate endogenous pain control are becoming well-recognized components of TTH pathophysiology, whereas anatomical studies have provided an understanding for the implication of cervical muscles in motor control. The increasing body of information related to sensorimotor adaptations in painful conditions should therefore shed light on functional limitations associated with primary headaches. Future research, however, is warranted to investigate the clinical aspect related to decrease in motor performance.

## Competing interest

The authors declare that they have no competing interests.

## Authors' contributions

AAM participated in the study design, acquisition of data, data analysis, and manuscript writing. VC participated in manuscript writing and revision. BM participated in study design, manuscript writing and revision. PS participated in manuscript revision. MD participated in study design, data analysis, manuscript writing and revision. All authors read and approved the final manuscript.

## Pre-publication history

The pre-publication history for this paper can be accessed here:

http://www.biomedcentral.com/1471-2474/15/179/prepub
